# Modeling properties of chromosome territories using polymer filaments in diverse confinement geometries

**DOI:** 10.1007/s10577-024-09753-z

**Published:** 2024-08-10

**Authors:** Negar Nahali, Mohammadsaleh Oshaghi, Jonas Paulsen

**Affiliations:** 1 Department of Informatics, Faculty of Mathematics and Natural Sciences, University of Oslo, Oslo, Norway; 2 Department of Biosciences, Faculty of Mathematics and Natural Sciences, University of Oslo, Oslo, Norway

**Keywords:** 3D genome modeling, Chromosome territories, Coarse-graining, Molecular dynamics

## Abstract

Interphase chromosomes reside within distinct nuclear regions known as chromosome territories (CTs). Recent observations from Hi-C analyses, a method mapping chromosomal interactions, have revealed varied decay in contact probabilities among different chromosomes. Our study explores the relationship between this contact decay and the particular shapes of the chromosome territories they occupy. For this, we employed molecular dynamics (MD) simulations to examine how confined polymers, resembling chromosomes, behave within different confinement geometries similar to chromosome territory boundaries. Our simulations unveil so far unreported relationships between contact probabilities and end-to-end distances varying based on different confinement geometries. These findings highlight the crucial impact of chromosome territories on shaping the larger-scale properties of 3D genome organization. They emphasize the intrinsic connection between the shapes of these territories and the contact behaviors exhibited by chromosomes. Understanding these correlations is key to accurately interpret Hi-C and microscopy data, and offers vital insights into the foundational principles governing genomic organization.

**Supplementary Information:**

The online version contains supplementary material available at 10.1007/s10577-024-09753-z.

## Introduction

Chromosomes are not randomly localized in a cell’s nucleus. Instead, they tend to occupy specific subdomains within the nucleus that are called chromosome territories (CTs) (Cremer and Cremer 2010; Cremer et al. 2006). CTs are known to be arranged radially within the nucleus. They typically possess a nonspherical structure which plays a significant role in cellular functions (Sehgal et al. 2014; Khalil et al. 2007). For instance, the irregular, nonspherical structure of CTs regulates gene expression by influencing interactions between distant genetic loci, impacts DNA repair mechanisms by affecting accessibility to damaged DNA (Branco and Pombo 2006), plays a role in accurate chromosome segregation during cell division, and contributes to the spatial organization within the nucleus, influencing vital cellular processes.

Chromosome territories (CTs) in the interphase nucleus of mammalian cells have been extensively studied but there are still open questions on their overall morphology, their internal properties and their structure-activity relationships (Fritz et al. 2019).

The polymeric nature of chromosomes suggests a tendency toward a mixed structure, contrary to the notion of distinct territories. Recent studies (Rosa and Everaers 2008; Kinney et al. 2018) show chromosomes fold into a non-equilibrium state, with no consensus on the time for equilibrium transition. In human chromosomes, this transition period is suggested to far exceed a cell’s lifespan (Jost et al. 2018), highlighting complex dynamics and hinting at equilibrium attainment being causative for CT existence.

To understand the folding of individual chromosomes within their chromosome territories (CTs), experiments utilizing ’chromosome conformation capture’ techniques (Dekker et al. 2002) provide a powerful methodology. These methodologies, including derivatives like 3C, 4C, 5C, and Hi-C (Han et al. 2018), facilitate the mapping of spatial contact frequencies. They enable the measurement of contact frequencies among pairwise proximal genome regions within and between chromosomes in the nucleus. Results from genome-wide 3*C* (Hi-C) experiments showed that the contact probability $P_{c}(l)$ between loci separated by a genomic distance *l* follows a power law of $p_c(l) \sim l^{-\alpha }$ (Barbieri et al. 2012; Belton et al. 2012). The exponent $\alpha$ is measured at $\sim 1.08$ for genomic distances between 0.5 and 7 Mbps, when averaged over all human chromosomes. Further analyses showed that, when considered individually, not all chromosomes exhibit the average human genome scaling behavior. For instance, chromosome 19 deviates significantly from the average value with $\alpha$ $\sim 1.30$. This suggests that each CT is governed by a distinct set of physical conditions, which needs further exploration to be understood properly.

Lately, polymer models (Bianco et al. 2018; Chiariello et al. 2016) have played a crucial role in improving our understanding of 3D genome folding and uncovering the outcomes of the experiments. Generic, large-scale features like the formation of chromosome territories or the decay of the average contact frequency $p_c(l)$ between two loci as a function of their genomic distance l ($p_c(l) \sim l^{-\alpha }$) have been well captured by simple topologically constrained self-avoiding polymers evolving in confined environment, the so-called crumpled or fractal polymer models (Mirny 2011; Rosa and Everaers 2008; Halverson et al. 2014).

Indeed, a promising strategy to better understand properties within chromosome territories is using confined polymer modeling, where chromosomes are confined within the CT boundaries. The characteristics of both flexible and rigid polymer chains confined within diverse cross-sectional rigid channels (e.g., rectangular or cylindrical shapes in 1D) are now established (Sheng and Wang 2001; Chen et al. 2004). Extensive utilization of scaling and mean field theories (Hsu and Binder 2013; Odijk 1983) has facilitated the exploration of both static and dynamic properties of these confined polymer chains.

Moreover, there have been numerous theoretical and experimental endeavors to unravel the potential interplay between different structural properties of the confined polymer and the confiner properties (Zhang et al. 2007; Zuo et al. 2019; Pastore et al. 2019). For instance, they investigate molecular mobility at interfaces, dynamic heterogeneities induced by surface properties, and dynamic decoupling in confined polymer systems, shedding light on the complexity of polymer dynamics under confinement.

The vast majority of numerical and analytical studies to date have focused on the case of a single long polymer chain in a spherical cavity (Gao et al. 2014; Chubak et al. 2022; Cacciuto and Luijten 2006). However, these models do not explain why territory shapes differ, nor how these shapes affect chromatin.

**Fig. 1:**
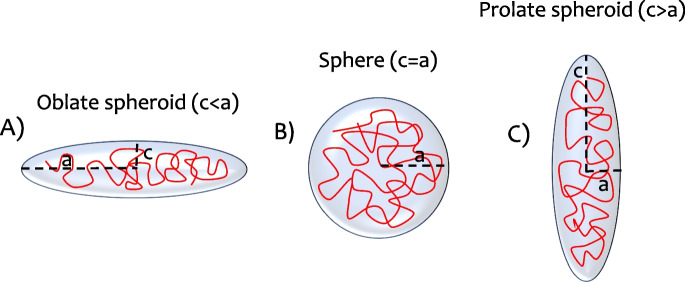
Schematic of a polymer in different confinement geometries: in oblate (*A*), two axes are equal, and the third is shorter ($c < a$); in spherical confinement (*B*), all axes are equal ($a = b = c$); in prolate (*C*), two axes are equal, and the third is longer ($c > a$)

Here, we shed light on the impact of different confinement shapes on the structural properties of the enclosed polymer. Considering three distinct confinement shapes (spherical, oblate, and prolate spheroids), we explore the role of the confinement geometry on $\alpha$. We show that at high densities, the spherical confinement displays the highest ${\alpha }$, whereas the most elongated prolate case exhibits the lowest ${\alpha }$, highlighting that contacts decay more rapidly in spherical case .Moreover, by varying the monomer density, and investigating how this affects the scaling of contact probability, we show that the decay of $p_c(l)$ is notably affected by the monomer density, indicating an opposite relation between density and the decay exponent. Utilizing Hi-C data, we additionally employ inverse modeling to show that the enrichment or depletion of contacts at specific lengths in different chromosomes depends on their average shapes. This dual approach unravels so far unreported relationships between confinement shapes and polymer folding.

## Results

### Direct modeling of the CTs: The shape of the confinement impacts intramolecular contact probabilities

To investigate the effect of confinement shape on a fixed-length polymer, we place a polymer consisting of $N=150$ monomers with diameter size $\sigma$ inside various confinement shapes. The polymer is represented using the Kremer and Grest polymer model (Kremer and Grest 1990), with each monomer representing roughly 1Mbp of chromatin, thus representing an averaged-sized chromosome in e.g. a human nucleus. The confinement shell is constructed using a series of monomers that have repulsive interactions with the interior polymer monomers (for more details, see Section 4.1).

To examine the effects of confinement shape, we defined sphere, oblate (flattened) and prolate (elongated) shapes (see Fig. 1). The degree of oblateness and prolateness was varied as well.

For the enclosed polymer, we considered monomer densities $\rho$ ranging from $\rho \sigma ^3=0.05$ to $\rho \sigma ^3=0.25$, mimicking realistic ranges seen in e.g. human cells (Ou et al. 2017). We also explore a wide range for the aspect ratio of the spheroids. The most elongated prolate spheroid has an aspect ratio of $\beta = \frac{c}{a} = 4$ where 2*c* and 2*a* are the length of the major and minor axes, respectively. On the other hand, the most compressed oblate spheroid has aspect ratio $\beta = 0.25$.

Our explored parameter space mimicks physiologically relevant conditions in the cell nucleus (Gürsoy et al. 2014). The monomer density $\rho$ depending on the species and the cell types can strongly vary (Eid et al. 2020; Li et al. 2024; Hawkins 2005). Given the substantial variability in chromatin volume concentration across different cell types, we explored a wide range for this parameter. Similarly, for the aspect ratio in CTs’ morphologies, which varies widely from spherical to ellipsoidal and even irregular shapes, we carried out independent runs of different confinement shapes and aspect ratios.

**Fig. 2:**
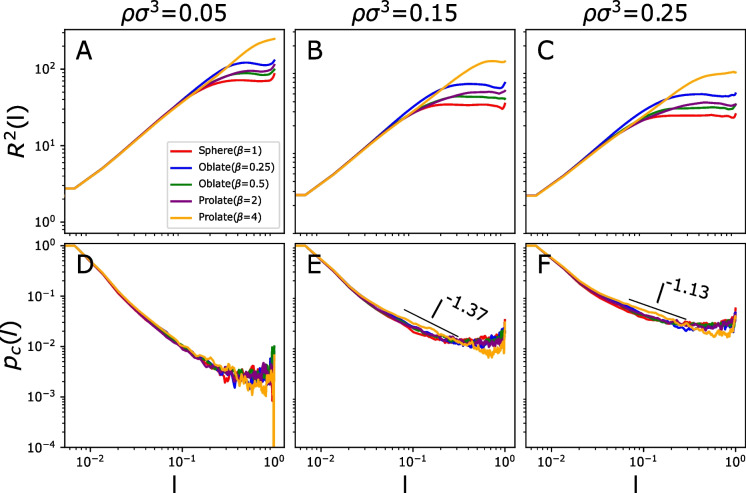
The upper panels (A-C) represent average-square internal distances $\langle R^2(l) \rangle$ between chain monomers at scaled length separation *l* at three different values of monomer density $\rho$. The lower panels (E-F) show contact frequencies $\langle p_c(l) \rangle$ between monomers at scaled length separation *l*. Results for different confinement geometries appear as distinct colour lines (see inset in panel A)

To analyze effects of territory geometry on monomer distances, we first computed the mean-squared Euclidean distance $<R^2(l)>$ between monomers located at contour length separation *l* along the polymer. The ensemble average $\langle ...\rangle$, is computed over the contour of the polymer and the last portion ($10\%$) of the simulations where the polymer has surely reached equilibrium. Results for $<R^2(l)>$ are summarized in Fig. 2 (A-C) from the most elongated prolate case ($\beta = 4$) to the most compressed oblate case ($\beta = 0.25$). Each panel represents a distinct value of the monomer density $\rho$. Results at different confinement geometries are represented by distinct colors. Note that, for the sake of easy comparison with the results of indirect modeling of CTs (“Inverse modeling of CT shapes: Prolate shapes dominate at medium length scales” section), we have rescaled l from 0 to 1.

As expected based on the theoretical scenario (Rubinstein et al. 2003), at short length scales outcomes for $\langle R^2(l)\rangle$ (Fig. 2(A-C)) show no dependence on geometry at any of the modeled densities. At large length scales, all the plots plateau except the most elongated prolate case at the lowest density ($\rho \sigma ^3=0.05$, Fig. 2(A)), as in this case the polymer can freely explore the longest axis of the confinement, which is much greater than the persistence length $l_p$. This situation only occurs in the most elongated prolate case, where one axis is much longer than the other two.

As shown in Fig. 2(C), for the scaled length *l* ranging approximately from 0.07 to 0.2 (corresponding to the range of 10-30 Mbp) and the highest density ($\rho \sigma ^3=0.25$), the end-to-end distance plots of the most extreme nonspherical confinement shapes (oblate [$\beta = 0.25$] and prolate [$\beta = 4$]) initially follow the measures for the spherical case but become larger towards the end of the range. Thus, within this range, in extreme prolate or oblate shapes monomers display a larger average end-to-end distance than in a spherical configuration.

For values of *l* beyond 0.2 ($>30Mbp$), end-to-end distances are consistently smaller for the spherical confinement, with the most extreme prolate case showing the highest difference. A similar trend, albeit less noticeable, is observed for lower densities ($\rho \sigma ^3=0.15$ Fig. 2(B) and $\rho \sigma ^3=0.05$ (A)). We also examined contact probability and mean-squared end-to-end distances for a larger polymer ($N=250$) at the highest monomer density ($\rho \sigma ^3=0.25$), showing comparable results (see Fig. S1). Interpretation of these results in relation to known 3D genome interactions in human chromosomes suggests that majorly A and B compartments and subcompartments (Ulianov et al. 2015) would be affected by these shifts, as their interactions are known to be at the scale of whole chromosomes and typically $>30Mbp$. Processes shaping TADs and sub-TAD structures, including DNA loop extrusion and enhancer-promoter interactions are likely less affected, at least directly, as these are typically found at scales $<1Mbp$ (Arensbergen et al. 2014).

Next, we studied the effect of geometry on the average contact frequencies, $\langle p_c(l) \rangle$, between polymer monomers at the scaled length *l* (see Fig. 2(E-F)). Two monomers *A* and *B* of diameter $\sigma$ inside a polymer chain are said to be in contact if their centre-to-centre spatial distance $r_{AB}$ is smaller than $r_{cutoff}=2\sigma$. Moreover, because of excluded volume effects from the LJ potential, two monomers can not be closer than the monomer size $\sigma$.

The contact probability $\langle p_c(l) \rangle$ of two loci separated by the scaled length *l* can provide information into the arrangement of the polymer depending on its scaling exponent. To this end, we study the scaling exponent $\alpha$ of the contact probability $p_c(l) \sim l^{-\alpha }$ between two sites separated by the scaled length *l* for all our cases. In our calculations, ${\alpha }$ is computed by standard best log-log fit of the corresponding $p_c(l)$ to a linear function. The fit range was limited to the range $0.07< l < 0.2$, because this is where the difference in behavior of different shapes appear while the plateau regime is not yet reached.

$p_c(l) \sim l^{-2.18}$ (Kang et al. 2015) is measured for unconfined self-avoiding walk (SAW). We expect that $p_c(l)$, in our confined cases, has a power-law behavior where the exponent $\alpha$ is dependent on both geometry and the density. We characterize the role of shape and density on $\alpha$.

When the density is low ($\rho \sigma ^3=0.05$), the exponent is ${\alpha } \sim 1.84$, which is the signature of the randomly folded (open) polymer (SAW model). For higher densities, the scaling exponent changes in a range depending on the confinement shape; we find the lowest value ${\alpha } = 1.13$ for the highest density and the most elongated prolate spheroid (see Table 1). At large length scales, the polymer shrinks into a compact mass manifested by a plateau of $p_c(l)$, where the exponent becomes ${\alpha } = 0$. This phenomenon arises due to the dominance of the polymer surface at large length scales, resulting in the absence of scaling behavior.

Overall, we find that the effective scaling exponent ${\alpha }$ slowly increases with decreasing $\rho$, reflecting a rather slow convergence to the asymptotic behavior expected from simple polymer scaling theory. At the lowest density (largest confinement size, $\rho \sigma ^3=0.05$), $p_c(l)$’s decay remains nearly unaffected by the confinement shape. When density increases, the largest ${\alpha }$ is always associated with the spherical confinement and the lowest with the most elongated prolate case. While the latter is more consistent with a crumpled globule, the former has more of self-avoiding walk-like features.

**Table 1:** Measured contact probability exponent (${\alpha }$) for all the confinement geometries at all densities

${\alpha }$	$\rho \sigma ^3=0.05$	$\rho \sigma ^3=0.15$	$\rho \sigma ^3=0.25$
Sphere	1.85	1.53	1.27
Oblate(${\beta }$ = 0.5)	1.85	1.47	1.24
Oblate(${\beta }$ = 0.25)	1.84	1.45	1.23
Prolate(${\beta }$ = 2)	1.86	1.46	1.21
Prolate(${\beta }$ = 4)	1.83	1.37	1.13

**Fig. 3:**
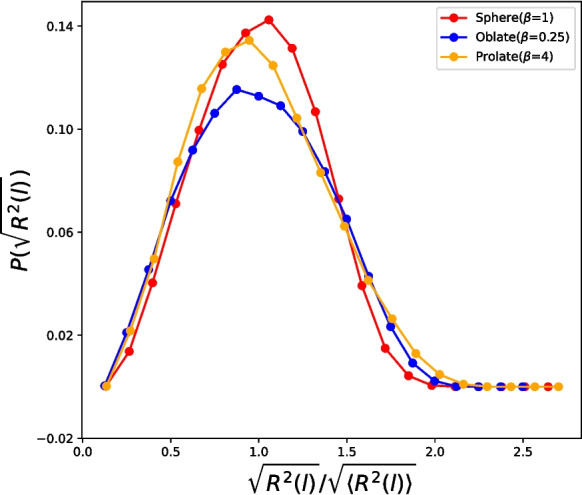
Distribution function of normalized end-to-end distances $P(\sqrt{R^2(l)}/\sqrt{\langle R^2(l) \rangle })$ at $l_{contour}=10\sigma (l = 0.07)$ for three different geometries at the highest density ($\rho \sigma ^3=0.25$). For the most elongated prolate case, the distribution is shifted toward smaller values of distances

In addition to understanding how contact probabilities decay, it is important to consider how end-to-end distances relate to these probabilities. As mentioned earlier, when the scaled length *l* is roughly between 0.07 and 0.2, $\langle R^2(l) \rangle$ is larger for the most elongated prolate case compared to the spherical confinement. Thus, on average, the monomers are more spread out in the extreme prolate case. On the other hand, the contact probability for the prolate case is higher than in the spherical case at this specific length (see Fig. 2(F)). In order to interpret this seemingly contradictory observation, we compute the normalized end-to-end distance probability distribution $P(\sqrt{R^2(l)}/\sqrt{\langle R^2(l) \rangle })$ at $l=0.07$ to understand changes when we switch between different shapes. Figure 3 highlights distribution differences among all the geometry shapes at the highest monomer density. $P(\sqrt{R^2(l)}/\sqrt{\langle R^2(l) \rangle })$ for the most elongated prolate case is shifted towards the lower end of the distribution and consequently results in a higher value for the contact probability compared to other geometries.

### Inverse modeling of CT shapes: Prolate shapes dominate at medium length scales

We then aimed to explore the relationship between the folding pattern of the polymer and the resulting shape it assumes using inverse modeling. To accomplish this, we extracted the interaction data of the human genome from available IMR90 Hi-C data (Consortium et al. 2012; Davis et al. 2018) and conducted simulations using Chrom3D (Paulsen et al. 2017)(for details, see Sections 4.4 and 4.6). Chrom3D is a 3D genome modeling computational tool, simulating the spatial arrangement of chromosome domains in relation to each other and their proximity to the nuclear periphery. In Fig. S2, we present the results of the approximated CT shapes derived from the simulations for chromosomes $1-22$. We use Principal Component Analysis (PCA) to identify the principal axes of the chromosomes. Subsequently, a spheroid equation is fitted to the monomers of the individual chromosomes to approximate the shape of each individual chromosome. In all chromosomes, the dominant shape category is prolate, with a minor presence of sphere. However, chromosomes 12, 15, and 18 exclusively exhibit prolate and oblate shapes. Notably, these are gene poor chromosomes, and have indeed been reported to correlate with more regular prolate shapes (Sehgal et al. 2014).

We then compared contact probabilities of chromosomes at different length scales by rescaling the x-axis to range from 0 to 1. This allowed for a standardized comparison of chromosomes, irrespective of their varying lengths.

To correct for the distance effect, where the chromosome length would influence the results, we divided the contact probabilities by an average value obtained by summing all the contact probabilities at corresponding lengths across all chromosomes. This normalization process effectively reduced the impact of distance-related variations in contact probabilities, enabling us to make meaningful comparisons between chromosomes with different lengths.

**Fig. 4:**
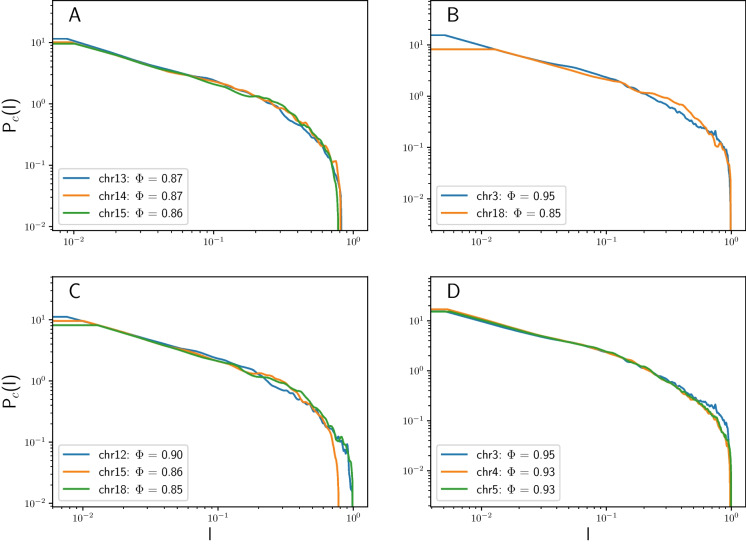
Comparison of normalized and rescaled contact probabilities among different chromosome groups. The legends display the chromosomes alongside their corresponding sphericity($\Phi$) values

We also normalized the surface area under the contact probability curves by counting the total number of contacts to accurately compare the relative contact enrichment or depletion patterns across different chromosome pairs (See Fig. 4).

To compare chromosomes based on their shapes, we measured their sphericity ($\Phi$), which quantifies the similarity of an ellipsoid to a sphere. The sphericity of a chromosome ellipsoid is calculated using the following formula in our analysis:

1 $$\begin{aligned} \Phi = \frac{\pi ^{\frac{1}{3}} \cdot (6 \cdot \text {volume})^{\frac{2}{3}}}{\text {surface\_area}} \end{aligned}$$

Volume and surface area are calculated based on the shape estimated through the fitting spheroid.The resulting $\Phi$ value ranges from 0 to 1, with higher values indicating a more spherical shape and lower values suggesting a more elongated or irregular shape.

In Fig. 4, we present comparisons of the contact probabilities for different groups of chromosomes. Based on $\Phi$, chromosomes 13, 14, and 15 exhibit low sphericity and a high contribution of elongated prolate shapes in their replicas. Consequently, the contact probability of these chromosomes decreases to zero before reaching the largest possible length scale (i.e., one on the x-axis) Fig. 4(A). This outcome aligns with the results obtained from direct modeling of confinement shapes in Fig. 2 where highly elongated prolate confinement had the lowest contact probability at large length scales ($l \sim 10^{0}$) among all shapes.

Comparing chromosome 3 (high $\Phi$) and chromosome 18 (low $\Phi$ and thus largely prolate) shows similar contact probabilities for short length scales ($10^{-2}< l < 10^{-1}$), due to low influence of confinement shapes. However, at larger scales ($10^{-1}< l < 10^{0}$), chromosome 18 dominates. This finding nearly mirrors the results from direct modeling, where the most elongated prolate case demonstrated higher contact probability at $l \sim 10^{-1}$ (Fig. 2(F)).

We then compared chromosomes 12, 15, and 18, all of which are aspherical based on $\Phi$. Among these three chromosomes, 15 and 18 exhibit the most extreme prolate cases, and as evident in Fig. 4(C), they dominate at medium length scales ($l \sim 10^{-1}$) compared to chromosome 12.

Based on $\Phi$, chromosomes 3, 4, and 5, all possessing high sphericity values, albeit with slightly different shape distributions. Indeed, the contact probability trends for chromosomes 4 and 5 are nearly identical (Fig. 4(D)). Chromosome 3 displays a higher contact probability at very large length scales( $l \sim 10^{0}$), while simultaneously having the highest contribution of spherical shapes among the three.

To ensure the robustness of our analysis across cell types, we did the same simulations using data from a human embryonic stem cell line (hESC). In Fig. S3, we illustrate the shape categorization of hESCs chromosomes. Similar to the observations in IMR90 cells, prolate shapes dominate in most chromosomes. In Fig. S4, we compare the contact probability patterns among different chromosome groups. Consistently, we observe analogous results to those found in IMR90 cells. Specifically, at medium length scales, prolate shapes exhibit predominant contact probabilities. For hESC cell lines, We also show the non-rescaled contact probability data for all the chromosomes in Fig. S5 to illustrate the raw data.

Our inverse modeling supports the observations from the direct modeling. We noticed that when looking at medium-length scales (around $l \sim 10^{-1}$), chromosomes with more elongated shapes had increased contact. On the other hand, at longer lengths (around $l \sim 10^{0}$), chromosomes with some spherical shapes or less elongated ones tended to have slightly higher contact. This emphasizes the crucial role of the confinement’s shape in determining how chromosomes fold.

## Discussion

Studying the impact of confinement shape on the folding of confined polymers is of particular significance for various biological systems. An illustrative instance of this phenomenon can be found with chromosomes, which reside within chromosome territories within the nucleus during interphase. Nevertheless, the precise implications of confinement shape on the folding and compaction of chromatin remain insufficiently understood. This challenge is enhanced by the substantial difficulty in effectively sampling lengthy, self-avoiding chromatin chains within the limited confines of the chromosome territories.

In this work, we present a Molecular Dynamics (MD) simulation study investigating the impact of diverse confinement shapes on the conformational behavior of individual chains. For simplicity, we focus on the behavior of a pure homopolymer. While our simplified system may not fully replicate the complexities of chromatin, we do observe significant variations in conformational outcomes across different confinement shapes at this level of complexity.

Moreover, our simulation results sample equilibrium conformations, which may not represent all chromosomes. Yet, considering that shorter to medium-length chromosomes to some extent likely have reached equilibrium, there is still partial equilibration and transferability of our results to realistic settings.

This study employs modeling of chromosome territories to analyze the effects of different confinement shapes on the folding behavior of the polymer. Furthermore, we also extract the shapes of chromosome territories (CTs) from Hi-C interaction matrices to compare contact probabilities. In the first scenario, which involves the direct modeling of confinement shapes, our analysis progresses from shape to folding, exploring how different shapes influence the folded state. In the latter scenario, we reverse the perspective, initiating from the folded state and working backward to understand how folding leads to the observed shapes of CTs.

Our results show that the spatial confinement of a polymer within different confinement shapes gives rise to distinct scaling relationships (${\alpha }$). $p_c(l)$’s decay strongly depends on the monomer density with an inverse relationship between confinement and the decaying exponent, the more polymer is confined, lower the ${\alpha }$. At the highest density, the largest ${\alpha }$ is always associated with the spherical confinement and the lowest with the most elongated prolate case.

We find that differences in the decay of the contact probability $p_{c}(l)$ between different shapes is associated with a clear change in the distribution of end-to-end distances $P(\sqrt{R^2(l)})$ (see Fig. 3). At a medium length scale ($l \sim 10^{-1}$), the shape of $P(\sqrt{R^2(l)})$ becomes less sharp and broadens for both oblate and prolate spheroids, that is, the chain and its ends experience greater conformational freedom. However, due to the reduction of chain conformational entropy, the probability of finding separations beyond the average are penalized. While for the spherical case, the decay is rapid resulting in a short-ranged decaying tail. The observed broadening from a more peaked to a more diffuse $P(R_{ee})$ is indicative of differences in contact rate decay between different geometries.

From our inverse modeling, we observed two phenomena that aligned with the findings from direct modeling. Firstly, at medium length scales (i.e., when $l \sim 10^{-1}$), chromosomes with a higher number of specially elongated prolate cases exhibited increased contact probability. Conversely, at larger length scales (i.e., when $l \sim 10^{0}$), chromosomes with some instances of spherical shapes, as well as not very elongated prolate cases, tended to have slightly higher contact probabilities.

Taken together, our results highlight the role of confinement geometry in determining the properties of chromosomes. A proper understanding of these relationships are needed to interpret data in chromatin biology correctly. Notably, since both end-to-end distance and contact probabilities are influenced by confinement shapes at and above medium length scales, which translates to $>10 Mbp$ on typical human chromosomes, we expect that properties like A- and B compartments and other large-scale genome folding patterns will be affected by confinement shape. Conversely, we expect processes like loop extrusion and enhancer-promoter looping to be largely unaffected from confinement shape alterations, at least directly, as these happen typically at $<1$ Mbp length scales.

## Supplementary Information

Below is the link to the electronic supplementary material.

Supplementary file 1 (pdf 417 KB)

## Data Availability

The data that supports the findings of this study are available upon request from the authors.

## Abbreviations

CTs: Chromosome territories
3C: Chromosome conformation capture
4C: Chromosome conformation capture-on-Chip
5C: Chromosome conformation capture carbon copy
Hi-C: High-throughput chromatin conformation capture
Mbp: Megabase pair
MD: Molecular dynamics
