# Supplementary material for: Modeling properties of chromosome territories using polymer filaments in diverse confinement geometries
Source: Chromosome Res. 2024 Aug 10;32(3):11. doi: 10.1007/s10577-024-09753-z (PMC11316705; doi:10.1007/s10577-024-09753-z)
Supplement: Supplementary file 1 — (pdf 417 KB) [file 10577_2024_9753_MOESM1_ESM.pdf]

1 Supplementary Material

1.1 Modeling of confinement shapes and interior polymer structure

Polymer model –

To model the polymer, we employ the well-known Kremer and Grest polymer model (Kremer and Grest, 1990). Excluded volume interactions between monomers (including consecutive ones along the contour of the polymer) are modelled by the shifted and truncated Lennard-Jones (LJ) potential:

$$U_{LJ}(r) = \begin{cases} 4\epsilon \left[\left(\frac{\sigma}{r}\right)^{12} - \left(\frac{\sigma}{r}\right)^{6} + \frac{1}{4} \right] & r \leq r_c \\ 0 & r > r_c \end{cases} , \tag{1}$$

where r denotes the separation between the monomer centers. The cutoff distance $r_c = 2^{1/6}\sigma$ is chosen so that only the repulsive part of the Lennard-Jones is used. The energy scale is set by $\epsilon = \kappa_B T$ and the length scale by σ, both of which are set to unity in our simulations. Consistent with that, in this work all quantities are reported in reduced LJ units.

Nearest-neighbour monomers along the contour of the polymer are connected by the finitely extensible nonlinear elastic (FENE) potential, given by:

$$U_{FENE}(r) = \begin{cases} -0.5kR_0^2 \ln\left(1 - (r/R_0)^2\right) & r \leq R_0 \\ \infty & r > R_0 \end{cases} , \tag{2}$$

where $k = 30\epsilon/\sigma^2$ is the spring constant and $R_0 = 1.5\sigma$ is the maximum extension of the elastic FENE bond.

In order to maximize mutual polymer interpenetration at relatively moderate chain length (Müller, Wittmer, and Cates, 2000) and hence reduce the computational effort, we have introduced an additional bending energy penalty between consecutive triplets of neighbouring monomers along the polymer in order to control polymer stiffness:

$$U_{bend}(\theta) = k_\theta \left(1 - \cos\theta\right) . \tag{3}$$

Here, θ is the angle formed between adjacent bonds and $k_\theta = 1\kappa_B T$ is the bending constant. With this choice, the polymer is equivalent to a worm-like chain with Kuhn length $\ell_K = 2\sigma$ (Auhl et al., 2003).

Confinement model –

The primary goal of the confinement model is to replicate the boundaries of the chromosome territories (CTs) within a cell nucleus. As CTs can take on various shapes, it is essential to formulate a method capable of accommodating these shape variations. To address this challenge, we developed an approach in which we placed a specific number of monomers on each selected shape. This number, denoted as M, represents the maximum quantity of equidistant monomers, each with a diameter size σ, that can be put on the surface of the confinement shape.

To ensure the even distribution of monomers across the selected surface, we implemented a systematic process. Our strategy involved sequentially adding these monomers to the surface. At each iteration, we calculated the center to center distance between the newly added monomer and its closest previously added neighbors, upholding a minimum distance of 1σ between all possible pairs of monomers. By following this rule, we prevented any overlap or clustering of monomers. At the lowest density ($\rho\sigma^3 = 0.05$), M varied from 800 to 1000 monomers respectively for spherical and prolate cases, while for the largest density ($\rho\sigma^3 = 0.25$), this range was between 300 and 400.

Upon successfully placing all the feasible points (monomers) on the surface, we created the confinement shell. This confinement model offers a versatile framework for accurately simulating the chromatin territory boundaries in various shapes.

We assume a Lennard Jones potential between the confinement monomers and the monomers of the polymer. The dynamics of the confinement monomers is set to zero so the shell is a rigid confinement.

1.2 Simulation details

The static and kinetic properties of the polymer is studied using fixed-volume and constant-temperature Molecular Dynamics (MD) simulations with implicit solvent. MD simulations are performed using the LAMMPS package (Thompson et al., 2022). The equations of motion are integrated using a velocity Verlet algorithm, in which all monomers are weakly coupled to a Langevin heat bath with a local damping constant $\Gamma = 0.5\tau_{MD}^{-1}$ where $\tau_{MD} = \sigma(m/\epsilon)^{1/2}$ is the Lennard-Jones time and $m = 1$ is the conventional mass unit for all the monomers. The integration time step is set to $\Delta t = 0.012\tau_{MD}$. Polymer solutions are simulated for the run time of $2 \times 10^9\tau_{MD}$.

1.3 Choice of initial configurations and check for equilibration

After fixing the position of the monomer walls on the desired confinement geometries and putting a polymer into confinement, we used MD runs with a soft (i.e. non-diverging), capped repulsive interactions between chain monomers. At the end of these preparatory runs, we turn on the force field mentioned in section 1.1 and the simulation initiates.

To confirm equilibration, we monitored the mean square displacement of the chain's center of mass. Equilibration was determined by identifying the point at which the mean square displacement reached a steady state, starting from different initial configurations. Unless otherwise stated, chain properties at equilibrium were always calculated on the last tenth of the corresponding trajectory.

1.4 Polymer interaction modeling

In the "inverse modeling of the confinement shapes" (CT shapes), we utilized Chrom3D (Paulsen, Sekelja, et al., 2017), a computational framework designed for reconstructing 3D genome structures efficiently using chromosome contact data from Hi-C experiments.

Mapping quality thresholded intact Hi-C contact matrices from IMR90 cells were retrieved from ENCODE (https://www.encodeproject.org/) with the accession ID ENCFF281ILS. Interacting contact domains in the Hi-C matrices were likewise retrieved from the accession ID ENCFF091WLU. Inter- and intra-chromosomal contact frequencies among all contact domains were extracted from the Hi-C matrices using Straw (Durand et al., 2016) at 1 Mbp resolution. Significant inter- and intra-chromosomal interactions were statistically distinguished using a noncentral hypergeometric (NCHG) distribution test (Paulsen, Rødland, et al., 2014). Utilizing the significantly interacting chromosomal domains, a total of 50 Chrom3D simulations were run with a nuclear occupancy of 0.15, at 1 Mbp resolution and 2 million simulation steps (Paulsen, Sekelja, et al., 2017). Inter-chromosomal contact vs. distance probabilities were calculated with cooltools (2C et al., 2022).

1.5 Analysis of end-to-end distances and contact probability for $N = 250$

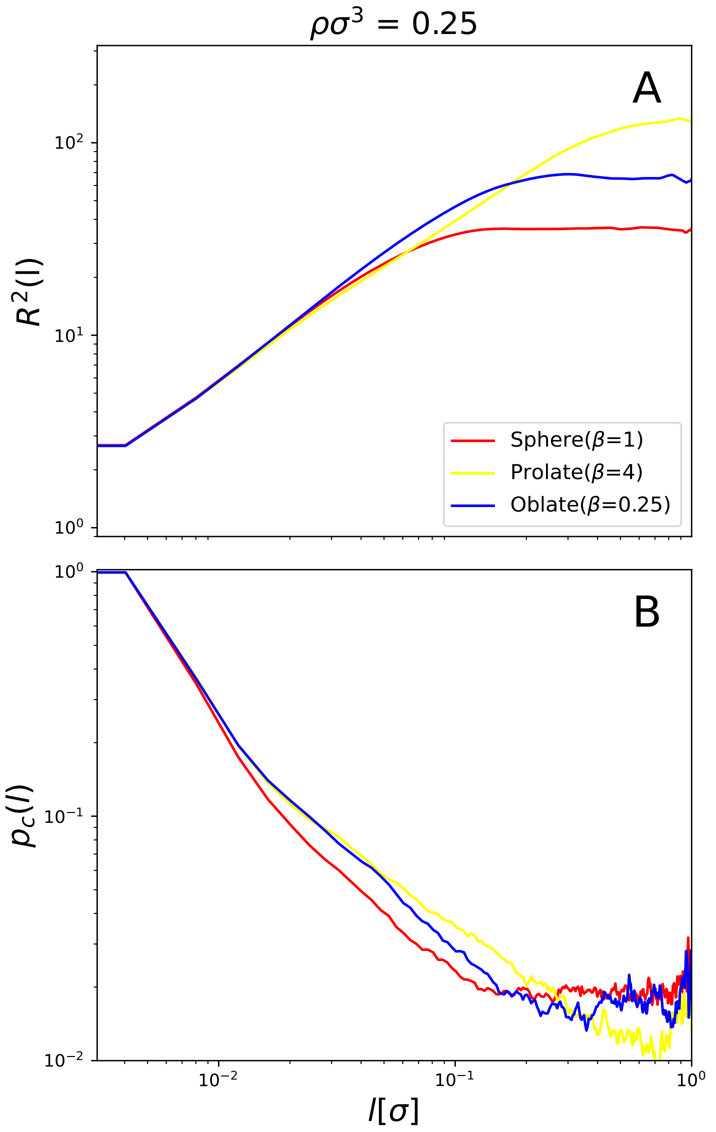

Figure S1: Panel (A) represents the mean-squared Euclidean distance $< R^2(l) >$ for a polymer with $N = 250$ monomers at $\rho\sigma^3 = 0.25$ at scaled length separation l. Panel (B) shows the contact probability $P_c(l)$ between monomers as a function of l.

1.6 CT shape analysis

In the inverse modeling, our objective was to determine the CT shapes of the chromosome configurations to enable meaningful comparison with the direct modeling of confinement shapes. We first employed PCA to determine the major axis of each configuration. Subsequently, we devised the following categorization criteria: If all three axes showed a similarity within 20% of the smallest axis, the configuration was designated as a sphere. Conversely, if two of the smallest axes exhibited a similarity within 20%, while the third axis demonstrated a difference larger than that, the configuration was classified as a prolate shape. Alternatively, if the two largest axes were within 20% difference of each other, and the smallest axis was substantially less than 20%, the configuration was identified as an oblate shape. The 20% threshold was chosen after considering the characteristics of our data and the nature of the system. This value represents a balance between accurate shape classification and accounting for the natural variability within the data. Any configuration that did not satisfy the conditions for these specific categories was deemed irregular and subsequently excluded from the analysis.

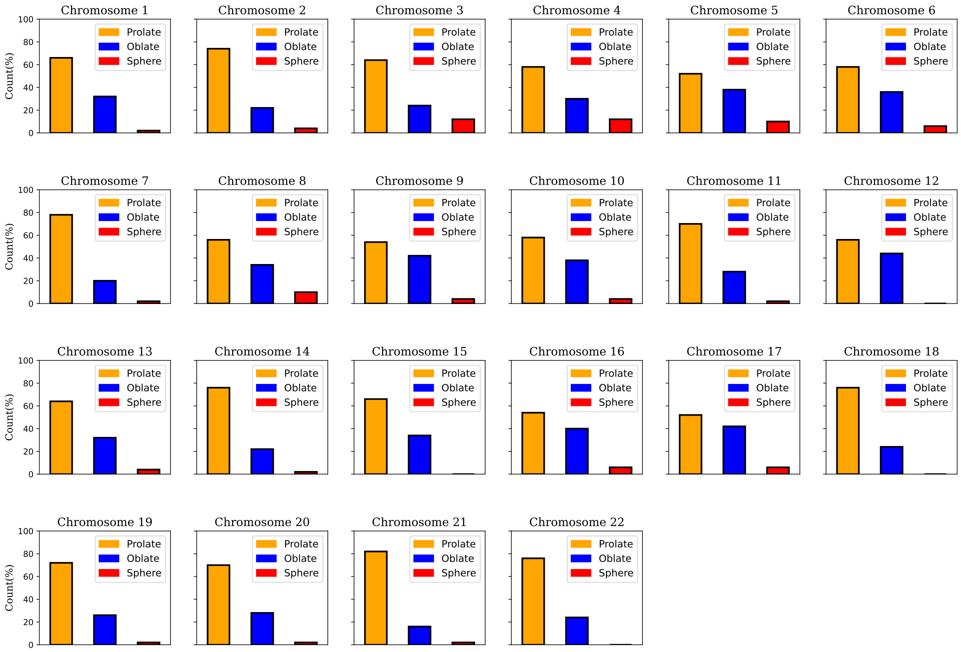

Figure S2: Analysis of the shape categories of human chromosomes 1-22. The shape categories are represented by three colors: prolate (orange), oblate (blue), and sphere (red). The counts of each shape category are shown as percentages.

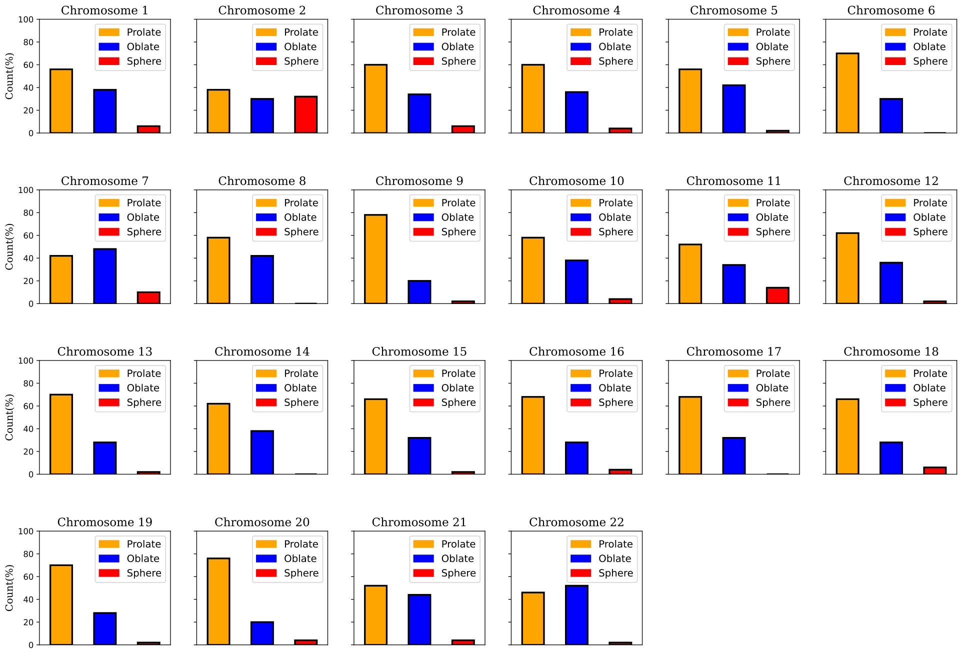

Figure S3: Analysis of the shape categories of human chromosomes 1-22 for Embryonic stem cells (ESCs). The shape categories are represented by three colors: prolate (orange), oblate (blue), and sphere (red). The counts of each shape category are shown as percentages.

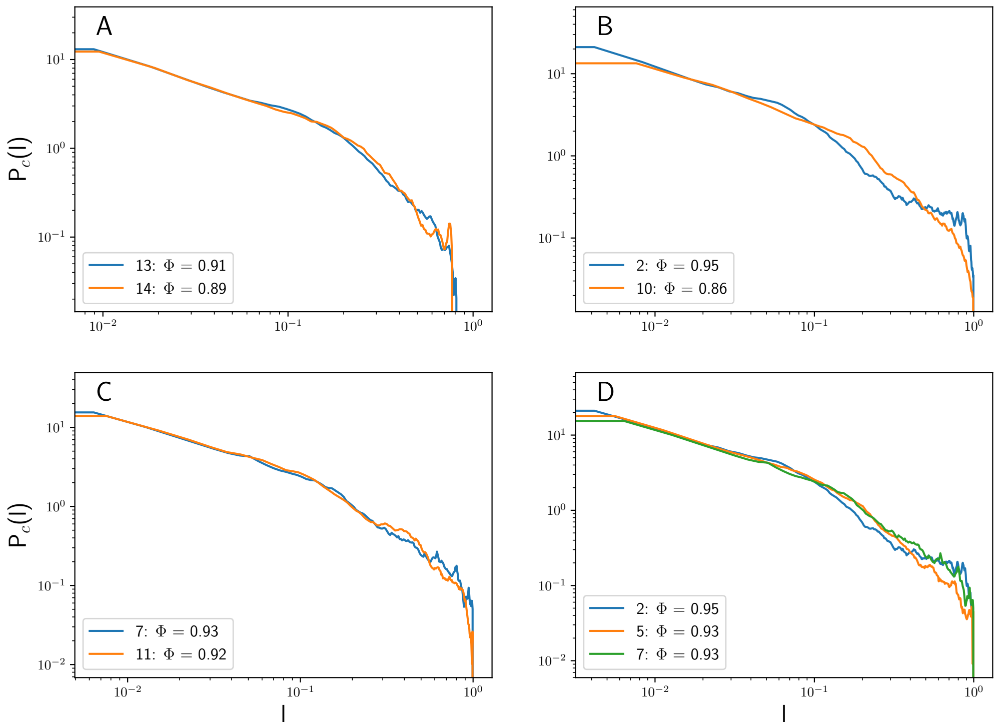

Figure S4: Comparison of normalized and rescaled contact probabilities among different chromosome groups in hESCs. The legends display the chromosomes alongside their corresponding sphericity(Φ) values.

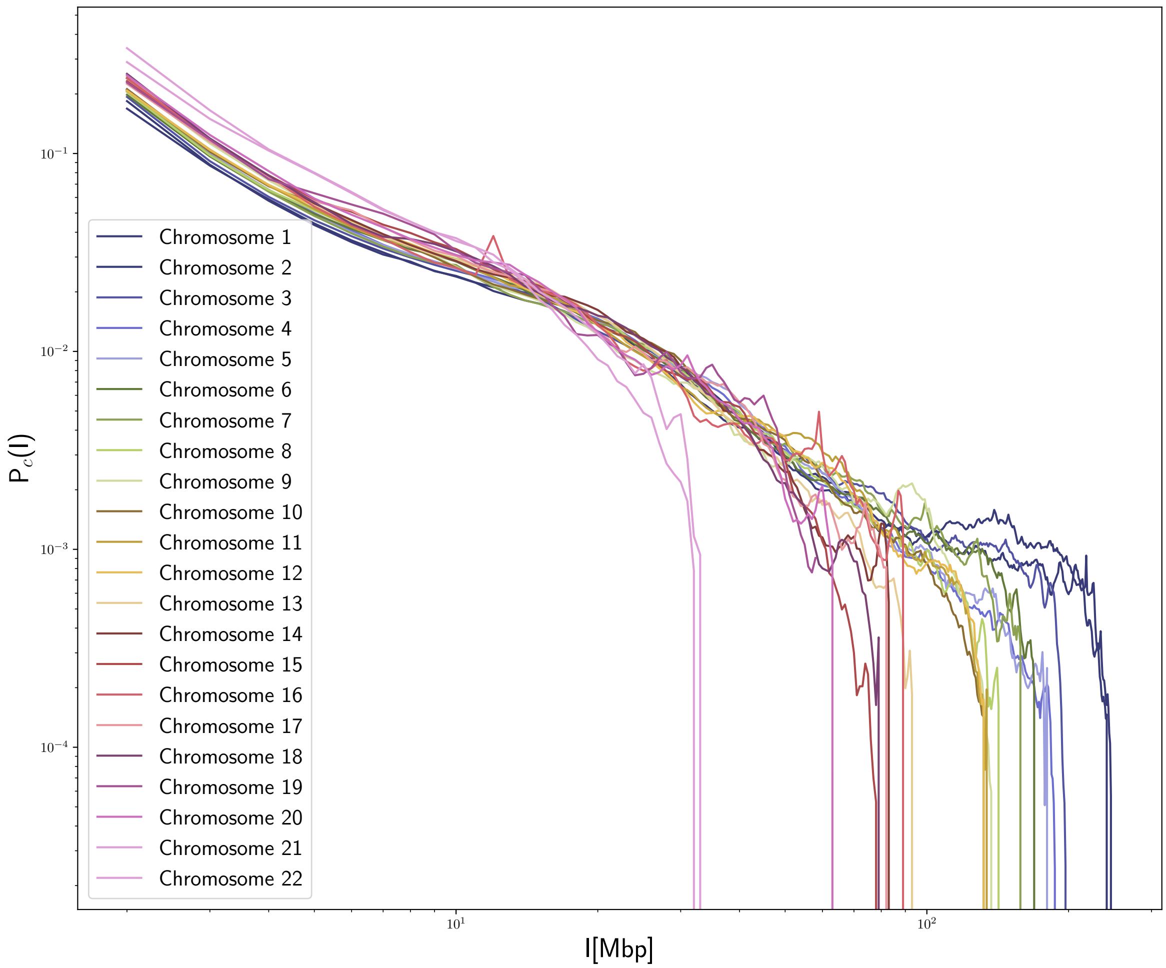

Figure S5: Chromosomal Contact Probability in hESCs: Each chromosome highlighted with a distinct color, illustrating intra-chromosomal interactions.
